# Modification of *Sargassum angustifolium* by molybdate during a facile cultivation for high-rate phosphate removal from wastewater: structural characterization and adsorptive behavior

**DOI:** 10.1007/s13205-016-0570-z

**Published:** 2016-11-22

**Authors:** Firozeh Saberzadeh Sarvestani, Hossein Esmaeili, Bahman Ramavandi

**Affiliations:** 1 Department of Chemical Engineering, Bushehr Branch, Islamic Azad University, Bushehr, Iran; 2 Environmental Health Engineering Department, Faculty of Health and Nutrition, Bushehr University of Medical Sciences, Bushehr, Iran

**Keywords:** *Sargassum angustifolium*, Phosphate adsorption, Modification, Algae–Mo, Real wastewaters

## Abstract

In this paper, a new and facile approach for molybdate loading in the brown algae of *Sargassum angustifolium* is introduced. The molybdate ions were entered into the algae body during a short cultivation to produce algae–Mo as a novel adsorbent for eliminating phosphate ions from synthetic and real wastewaters. Results of the surface analysis showed that molybdate loading onto the algae was successfully performed. Herein, basic variables, such as initial solution pH, adsorbent dosage, contact time, phosphate concentration, and temperature, were investigated in detail to assess the phosphate adsorption performance of algae–Mo. The pseudo-second-order kinetic model fitted our acquired experimental kinetic data most appropriately, in comparison to the use of a pseudo-first-order model. The Langmuir model appeared to fit the adsorption data more desirably than that of Freundlich and Dubnin–Radushkevich models, with a maximum phosphate adsorption capacity of 149.25 mg/g at 25 °C. The finding of the thermodynamic study revealed that the phosphate adsorption onto algae–Mo was spontaneous, feasible, and endothermic in nature. The study on Mo^2+^ ions leaching strongly suggested that the risk of Mo^2+^ leakage during phosphate adsorption was negligible at a wide pH range of 3–9. The adsorption efficiency attained was 53.4% at the sixth cycle of reusability. Two real wastewaters with different qualities were successfully treated by the algae–Mo, suggesting that the algae–Mo could be ordered for practical wastewater treatment.

## Introduction

Phosphate (PO_4_^3−^) is a basic material for many industries, including beverages, fertilizers, detergents, pharmaceuticals, paints, and corrosion inhibitors. Excess amounts of wastewater phosphate discharged into water reservoirs, such as streams, rivers, and lakes, can cause algal bloom and eutrophic conditions. To control such adverse effects in water bodies and to maintain good effluent quality, removal of the excessive phosphate in wastewaters is required. This will ensure that the phosphate concentration in effluent from wastewater treatment plants, runoff, and subsurface drainage from agricultural land and urban areas, as well as domestic septic systems does not exceed 0.1 mg/L, a concentration above which eutrophication is likely (Johir et al. 2015).

Although chemical and biological methods are most commonly used for phosphate removal, adsorption processes have attracted increasing attention, with the primary benefits of being effective, best suited for low levels of phosphate, and favorable to phosphate recovery (Nguyen et al. 2014a). So far, numerous adsorbent materials have been used for phosphate removal, such as sugarcane bagasse (Zhang et al. 2011), soybean milk residues (Nguyen et al. 2013), granular date stones (Ismail 2012), iron/zirconium-loaded okara (Nguyen et al. 2014a), zirconium-loaded MUROMAC (Biswas et al. 2008), and bayoxide-E33 (Lalley et al. 2016) with the maximum adsorption capacity of 1.10–131.77 mg/g. Thus, it is concluded that research into finding an efficient adsorbent with higher capacity for phosphate removal is still required. In this context, Nguyen et al. (2014b) recently reviewed methods of thermal, chemical, and steam activation, along with sulfate coating and metal loading, for modification of phosphate adsorbents. They pointed out that among these methods, metal loading adsorbents have main advantages, such as facile operation, high efficiency, and selectivity toward phosphate. Based on the published literature (Carvalho et al. 2011; Mallampati and Valiyaveettil 2013; Wang et al. 2012), the metals or cations were loaded on adsorbents after preparing a simple biochar of the adsorbent precursor. Preloading the biochar/adsorbent with metals reasonably improved its phosphate adsorption capacity (Wang et al. 2012) which could be attributed to the fact that metal contents are cross-linked to adsorbent leading to enhanced adsorption. The main challenge for this method is due to metal leaching from metal-amended adsorbents during water/wastewater treatment. Accordingly, the use of this type of amended adsorbents for phosphate removal on a large scale does not seem to be a feasible proposition. Therefore, more effort should be devoted toward developing a new method for metal loading on adsorbents to produce metal-amended adsorbents with higher adsorption capacity and without any metal leaching. Metal leaching from the adsorbent would increase the toxicity of the treated effluent.

Based on this, in a new approach we amended the living algae during a facile cultivation. The brown algae of *Sargassum angustifolium* (which could be abundantly found in marine water in all seasons) was chosen and modified by molybdate for enhancing phosphate adsorption. Molybdate is usually applied for phosphate measurement in aqueous solutions (Federation and Association 2005) as sequestrating agent. As we know, the natural metal content of plants and algae (Singh et al. 2016) is leached during digestion in a very acidic condition for environmental assessment and evaluation of the metal content of plants and algae. Therefore, if the algae are naturally amended by a given metal during their growth, the metal leaching during water/wastewater treatment will be hard and the treated solution is safe.

The main research aim herein was to modify *S. angustifolium* by molybdate during living and use its biochar to remove phosphate from aqueous solution and real wastewater, which, to the best of authors’ knowledge, has not been reported.

## Materials and methods

### Chemicals

All chemicals used, such as sodium hydroxide (NaOH, 98%), hydrochloric acid (HCl, 37%), ammonium molybdate tetrahydrate [(NH_4_)_6_Mo_7_O_24_·4H_2_O, 99.98%], and sodium dihydrogen phosphate anhydrous (KH_2_PO_4_, 99.99%), were of analytical reagent grade and purchased from Merck Co., Ltd. (Germany). Work solutions were prepared by doubly distilled water.

### Sampling, cultivation, and modification of *S. angustifolium*

The algae mass was taken in November of 2015 from the northern coast of Persian Gulf, Bushehr, Iran. According to the Iranian National Institute for Oceanography and Atmospheric Science, the harvested algae belonged to *S. angustifolium* specie. The collected algae were first washed with seawater to wash out the debris and then shipped to a laboratory by a 20-L container during 20 min. A glass reactor with a total volume of 40 L equipped with an air pump (0.5 L air/min) for the purpose of aeration was used for culturing *S. angustifolium*. 30 L of the seawater was poured into the reactor and then 4 g molybdate was added to it. After that, 600 g of *S. angustifolium* biomass was put into the reactor. No other material was added as a carbon or energy source during cultivation. The average intensity of 2500 Lux at the surface level of water and air temperature of 25 ± 1 °C were fixed for algae growth. The algae were grown under light/dark cycle 12/12 for eight consecutive days. After that, the algae biomass was picked up. The algae were fast growing as their weight was increased by about 9%. The algae biomass was washed with running tap water and distilled water and then dried in an oven at 150 °C for 2 h. The dried mass was ground and passed through an American Society for Testing and Materials (ASTM) sieve (mesh no. 20) to obtain uniform size particles (850 µm). This prepared powder was used in tests as modified algae by molybdate and namely “algae–Mo.” Equivalently, a portion of the collected algae from the Persian Gulf without any amendment process was dried, ground, and sieved to obtain an “unmodified algae” mass. The unmodified algae were used to explore the synergy effect of algae and molybdate. All experiments in this research were done from one single harvest and culture.

### Batch adsorption tests

The adsorption tests of phosphate ions were performed in batch mode. Phosphate stock solution (1 g/L) was made by dissolving 1.4329 g KH_2_PO_4_ in 1000 mL doubly distilled water. The method of “one parameter at the time” was used for optimization experiments. The various parameters designed were as follows: pH (3, 4, 5, 6, 7, 8, and 9), initial phosphate concentration (50, 70, and 100 mg/L), contact time (3, 5, 10, 20, 40, 60, and 80 min), algae–Mo dose (2, 5, 10, 15, and 20 g/L), and temperature (20, 25, 30, and 40 °C). During the optimization tests, the solution temperature and shaking rate were fixed at 25 °C and 120 rpm, respectively. The optimization was initiated by a pH test. To do this, 100 mL of phosphate solution containing 50 mg/L concentration was poured into 250-mL Erlenmeyer flasks. The initial solution pH was changed using HCl or NaOH (0.1 M) to the desired level; then, the amount of 10 g/L algae–Mo was added to the flasks. At the time of contact (60 min), the suspension was passed through a 0.45-μm filter paper and the phosphate concentration of filtrate was analyzed. The next parameter for optimization was of algae–Mo dose, which was tested under condition of pH 5, phosphate concentration 50 mg/L, and contact time 60 min. The effect of contact time and initial phosphate concentration was simultaneously tested at an adsorbent dose of 10 g/L and pH 5. The phosphate adsorption (PA) and the amount of phosphate adsorbed onto the algae–Mo, *q*_e_ (mg/g), were calculated as follows:

1 $${\text{PA}}_{{}} = \frac{{C_{\text{i}} - C_{\text{f}} }}{{C_{\text{i}} }} \times 100,$$

2 $$q_{\text{e}} = (C_{\text{i}} - C_{\text{e}} )\frac{V}{M},$$

where *C*_i_ (mg/L), *C*_f_ (mg/L), and *C*_e_ (mg/L) are the initial, final, and equilibrium concentrations of phosphate ion, respectively. *V* (L) is the volume of the working solution and *M* (g) the mass of algae–Mo.

The kinetics of phosphate adsorption onto the adsorbent prepared from *S. angustifolium* was assessed for three initial phosphate concentrations at optimal conditions. This test was conducted at various times between 3 and 80 min and pH 5. At the end of each test, the suspension was analyzed.

Tests to study the adsorption equilibrium were carried out by adding a given mass of 1 g algae–Mo into a series of Erlenmeyer flasks, containing 100 mL of various phosphate concentrations (10–100 mg/L) and constant pH of 5. Flasks were then stirred for 360 min at a constant temperature (25 °C) to attain equilibrium, after which the phosphate concentrations of the suspension were analyzed.

The thermodynamics of phosphate adsorption onto algae–Mo was assessed using adsorption tests done by an Erlenmeyer flask, containing 100 mL of 50 mg/L phosphate with pH 5 into which amounts of 1 g adsorbent were added and shaken at a fixed rate in a shaker incubator (Parsazma, Iran). The test was conducted at various temperatures between 20 and 40 °C, and after reaching the equilibrium the suspension was filtered and then analyzed.

The algae–Mo was regenerated for seven consecutive cycles using sodium hydroxide (1 M NaOH) solution. The regenerated algae–Mo was then subjected to phosphate adsorption under the identical experimental conditions of pH 5, initial phosphate concentration of 50 mg/L, contact time 60 min, and solution temperature 25 °C.

The potential of algae–Mo adsorbent in field conditions was tested with two bulks of samples. Sample 1 was provided from an effluent of primary sedimentation tank in the wastewater treatment plant of the Persian Gulf hospital, Bushehr, Iran. Sample 2 was collected from an urban wastewater before entrance to the Persian Gulf. This urban wastewater was naturally treated by a Canebrake which is beside the Persian Gulf, Bushehr coast. The main characteristic of sample 1 was its higher content of organic matter than sample 2. Sample 2 had a high chemical content. In other words, samples 1 and 2 were selected for applicability of algae–Mo for treating wastewaters with high organic and high total dissolved solid (TDS) value, respectively.

All tests (including experiment and control) were done twice for an experimental run to ensure reproducibility of the findings, and the average number of the measurements was considered for data analysis. The blank test (test without algae–Mo) was done to control the interferences of the impurity of the used material and test conditions in the adsorbate removal.

### Analytical methods

The pore diameter of algae–Mo was calculated from the adsorption branch of the isotherm using the Barrett–Joyner–Hallenda (BJH) method. The total pore volume of algae–Mo was evaluated from the adsorbed nitrogen amount at a relative pressure of 0.9925. The adsorbent specific surface area was estimated using the Brunauer–Emmett–Teller (BET) method with an analyzer of surface area (Micromeritics ASAP 2000, USA). The examination of surface morphologies of fresh and used algae–Mo samples was done by scanning electron microscopy (SEM, Sirion from FEI). The functional groups on the surface of the adsorbent before and after phosphate adsorption was recognized using a Fourier transform infrared spectroscopy spectrometer (FTIR, NICOLET 5700-FTIR) in the range of 400–4000 cm^−1^. The elemental composition of fresh and used algae–Mo was achieved by energy-dispersive spectroscopy (EDS, Horiba EX-250, Japan). The phosphate concentration was measured using the DR-2800 kit (Hach Lange). The parameter of pH of zero-point charge (pH_zpc_) was measured similar to a method reported elsewhere (Fooladvand and Ramavandi 2015). The pH of the working solutions was analyzed using a pH meter (Sense Ion 378, Hack). The intensity of illumination was measured by Hanna Instruments HI 97500. Molybdate concentration was analyzed using ICP-MS (PerkinElmer Elan DRC II). Other required parameters were analyzed according to the method presented in standard methods for the examination of water and wastewater (Federation and Association 2005).

The wet weight of algae was obtained by the analytical balance (HR120, Metler Toledo) after centrifugation at 500*g* for 5 min. By centrifuging at this low rate, it was found that *S. angustifolium* cells did not burst or suffer damage when examined under a microscope.

## Results and discussion

### Surface analyses

**Fig. 1:**
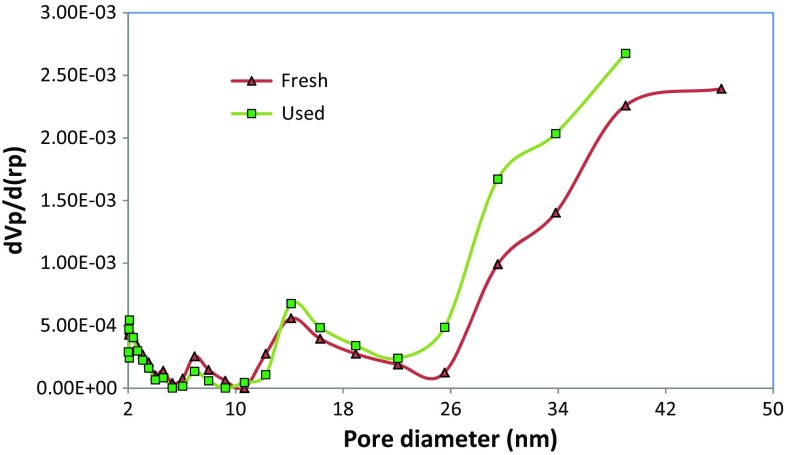
Pore diameter distribution of the fresh and used algae–Mo particles

**Fig. 2:**
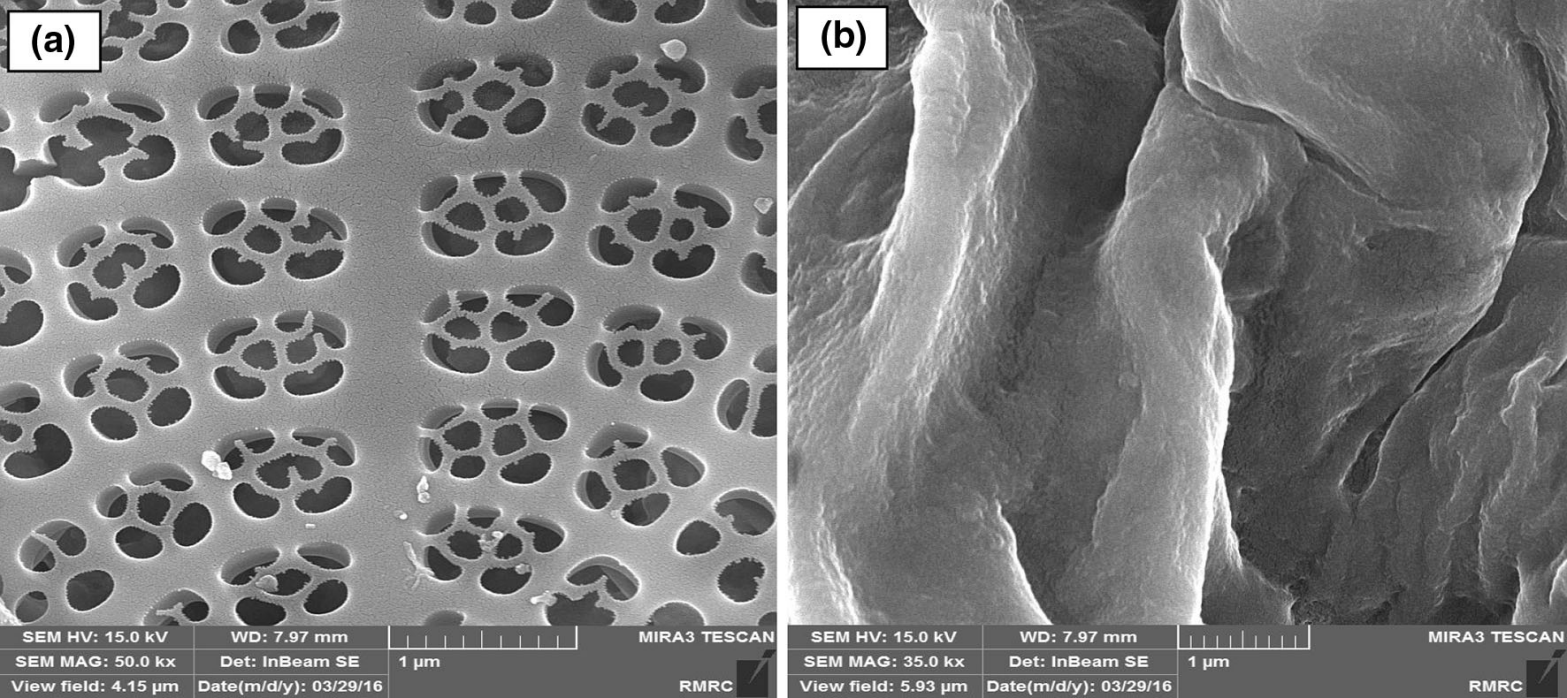
SEM images of algae–Mo **a** before and **b** after phosphate adsorption

**Fig. 3:**
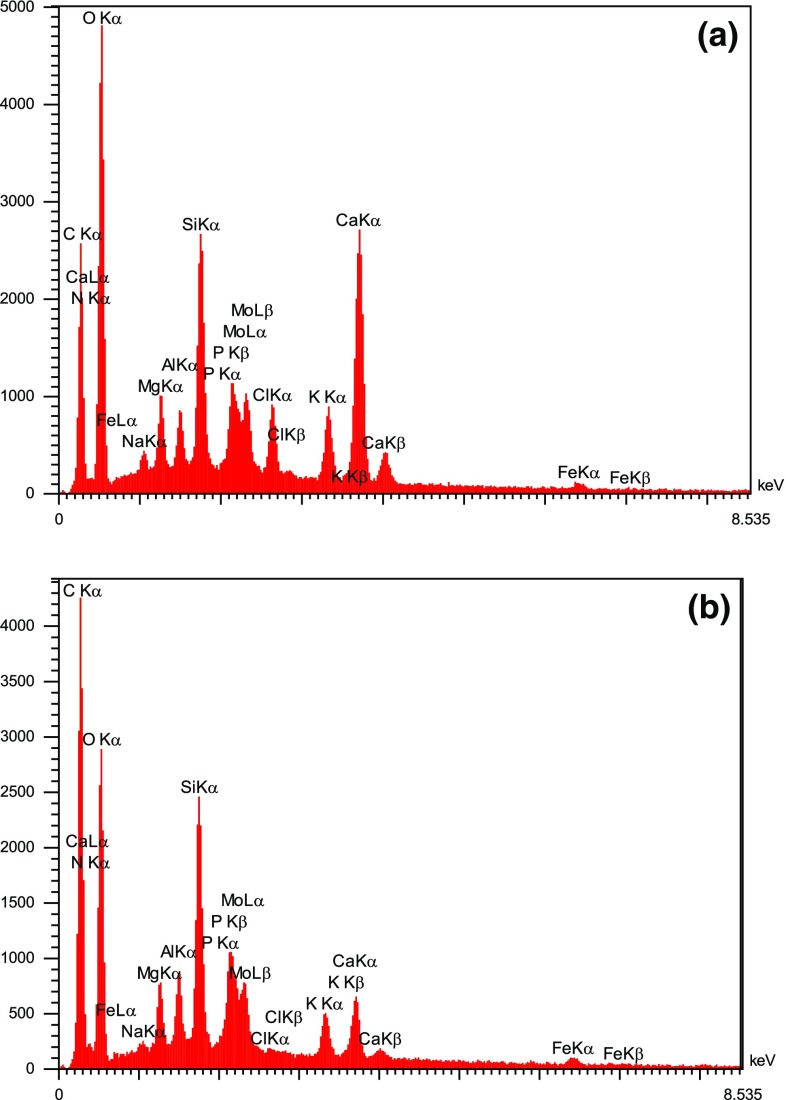
EDS spectrum of algae–Mo **a** before and **b** after phosphate adsorption

Surface analysis showed that algae–Mo particles had a BET multipoint surface area of 1.44 m^2^/g and a total pore volume of 0.0497 cm^3^/g. Because the BET surface area of the adsorbent is relatively low, the functional groups may have a more important role than particle surface area (Ramavandi et al. 2016). The pH_zpc_ of algae–Mo was obtained to be 5.4, signifying a negative surface charge for a working solution pH greater than 5.4 and a positive surface charge for a solution pH below 5.4. According to Fig. 1, the pore sizes of the modified algae before and after phosphate removal falls within the range of 2–50 nm, indicating that the algae–Mo adsorbent was a mesoporous type. The surface structures of the fresh and phosphate-loaded algae–Mo particles imaged at the same magnification are displayed in Fig. 2. As shown in Fig. 2a, the fresh adsorbent was a porous and smooth surface material. Figure 2b depicts algae–Mo after adsorption, indicating that phosphate ions were adsorbed evenly on the surface of algae–Mo. EDS spectra of adsorbent before and after phosphate adsorption (Fig. 3a, b) confirmed this observation. The results of elemental analysis of the algae–Mo for phosphate adsorption are presented in Table 1. As seen from Table 1, it is obvious that the algae were successfully modified by the molybdate element and the phosphate ion adsorbed by algae–Mo. The presence of phosphate on the adsorbent (~13.5 wt%) proved that the phosphate removed from the wastewater had been adsorbed onto the algae–Mo. On a closer look at Table 1, it is clear that only 0.04% of Mo was lost during the adsorption, indicating the good stability of the algae–Mo adsorbent.

**Table 1:** Results of EDS elemental analysis of algae–Mo for phosphate adsorption

Element	Value (wt%)	
	Before adsorption	After adsorption
C	28.94	34.11
N	2.92	2.81
O	47.87	38.36
Mg	0.77	0.53
Al	0.52	0.52
Si	3.29	3.20
P	0.05	13.52
Cl	1.42	0.05
K	1.95	1.15
Ca	8.27	1.81
Fe	0.43	0.41
Mo	3.57	3.53
	100.00	100.00

**Fig. 4:**
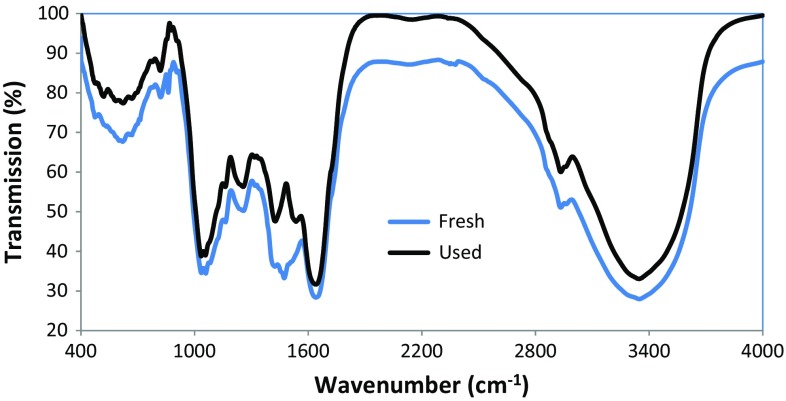
FTIR spectrum of the fresh and used algae–Mo

The FTIR pattern for the algae–Mo before and after the adsorption of phosphate is presented in Fig. 4. The presence of an intense and wide band in the 3374 cm^−1^ region and a peak at 2919 cm^−1^ for the metal hydroxide before adsorption may be due to the stretching vibration form of lattice water and hydroxyl groups (Johir et al. 2016). Thus, the FTIR results are in accordance with those obtained by the EDS spectra. A peak at 1483 cm^−1^ (O–H bending vibration) is due to coordinated water molecules, and that at 1268 cm^−1^ (O–H bending vibration) indicated surface hydroxyl group on the algae–Mo surface (Wang et al. 2014). Lu et al. (2014) pointed out that the bands at 800 and 400 cm^−1^ could be ascribed to the metal oxide (here, MoO_2_). As depicted in Fig. 4, the intensity of the described peaks was significantly weakened after phosphate adsorption, suggesting the possible role of functional groups in the adsorption process.

Compared to published research (Jiang et al. 2016; Ma et al. 2014), the *P* content of the used adsorbent (see Table 1) is relatively high. Therefore, the used adsorbent can be reused either as a compound fertilizer or as a conventional fertilizer superaddition in agriculture. However, further evaluation of the application potential of the algae–Mo, its fertilizer cost-effectiveness, and environmental and health impact should be considered.

### Effect of pH

**Fig. 5:**
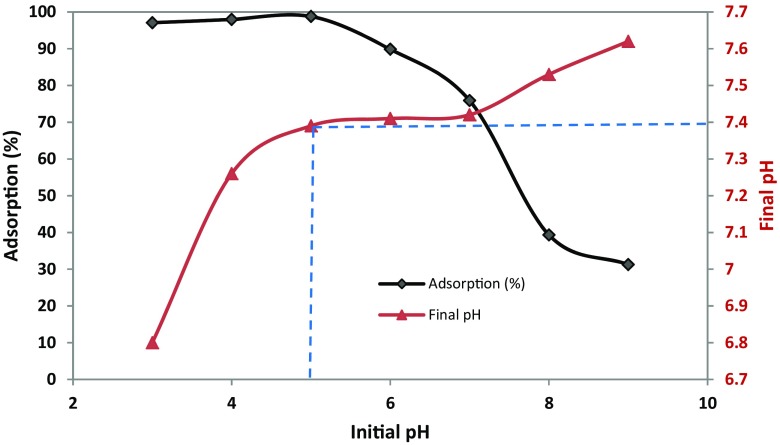
Effect of initial solution pH on the phosphate adsorption (adsorbent dose 10 g/L, phosphate concentration 50 mg/L, contact time 60 min)

**Fig. 6:**
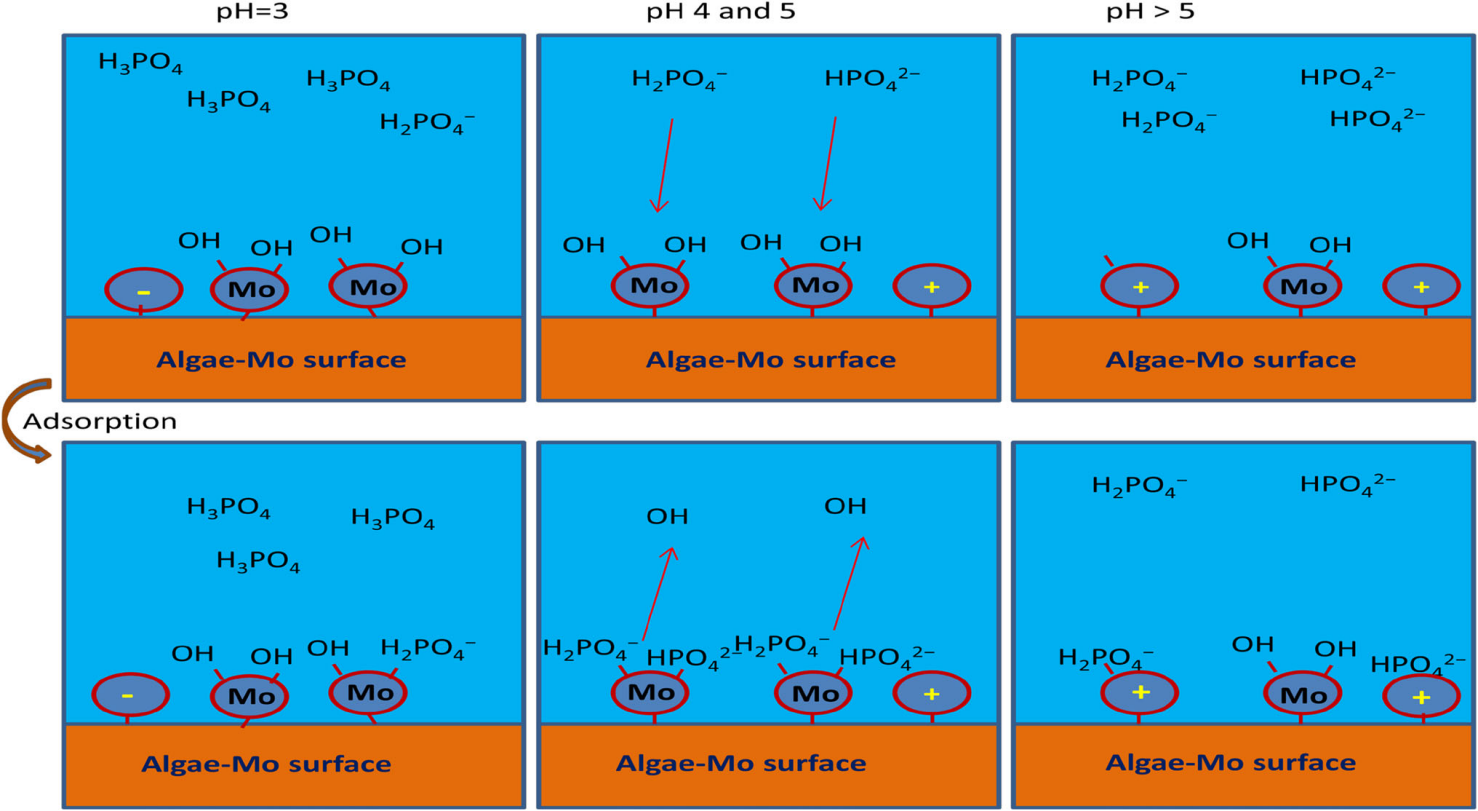
Proposed mechanism of phosphate adsorption onto the algae–Mo surface

The solution pH effect on the phosphate adsorption by algae–Mo particles is depicted in Fig. 5. The phosphate adsorption onto the algae–Mo was evidently pH dependent. At the more acidic pH status, the adsorption removal just increased very slightly from pH 3 to 5, while it dropped much faster with the pH increase after pH 6. Similar behavior by other researchers (Lu et al. 2015; Lǚ et al. 2013; Wang et al. 2014) had also been observed for various adsorbents. The highest phosphate adsorption removal of 98.76% was observed when the solution pH value was 5. The pH effect could be defined by pH_zpc_ and the dominant species of phosphate ion existence in the solution. Under the solution pH range studied (pH 3–9), H_2_PO_4_^−^ and HPO_4_^2−^ were the dominant species of phosphate in the solution at pH 5–9 and H_3_PO_4_ in the main form of phosphate at more acidic solution (here, pH < 5). The pH_zpc_ of algae–Mo was about 5.4 and its surface was negatively charged at pH < 5.4, which repulsed the negatively charged phosphate ions and expected to achieve very low phosphate removal. However, at low solution pH (i.e., pH < 5), the phosphate adsorption was attained around 97%, which is unpredicted. The unexpected and higher adsorption removal achieved in the acidic pH values may be described by the ligand-exchange mechanism. Similarly to other metal oxides (Huang et al. 2014; Johir et al. 2016; Liu et al. 2016), the embedded molybdate in algae–Mo forms hydroxide when in the aqueous solution, specifically attracting phosphate via the ligand-exchange mechanism, as exhibited in Fig. 6. The ligand-exchange mechanism involved in the phosphate adsorption process by using algae–Mo can be further supported by monitoring the pH alteration during the adsorption process, as shown in Fig. 5. After adding 10 g/L of algae–Mo to a solution with initial pH of 5, the pH value finally reached the neutral zone (7.4). Attaining a lower level of phosphate adsorption among acidic pH values is because of phosphoric acid (H_3_PO_4_) formation, which was not as active as H_2_PO_4_^−^ and HPO_4_^2−^ in the chemical adsorption (Ye et al. 2015). These explications are also depicted in Fig. 6 for better understanding. For further tests, the optimum pH for phosphate removal by algae–Mo was 5. The low percentage of phosphate removal at pH > 5 could be explained by the competition between the hydroxide anion and phosphate ions for active adsorption sites. In general, two major advantages of the algae–Mo will increase its likelihood of being used practically in wastewater treatment: (I) attaining acceptable phosphate removal efficiency in the wide range of pH (3–7) and (II) raising the treated solution pH to the neutral region; thus, neutralization the effluent pH before entering the water bodies is not required. This will lead to lowering the total cost of phosphate treatment using algae–Mo.

### Effect of adsorbent dose

**Fig. 7:**
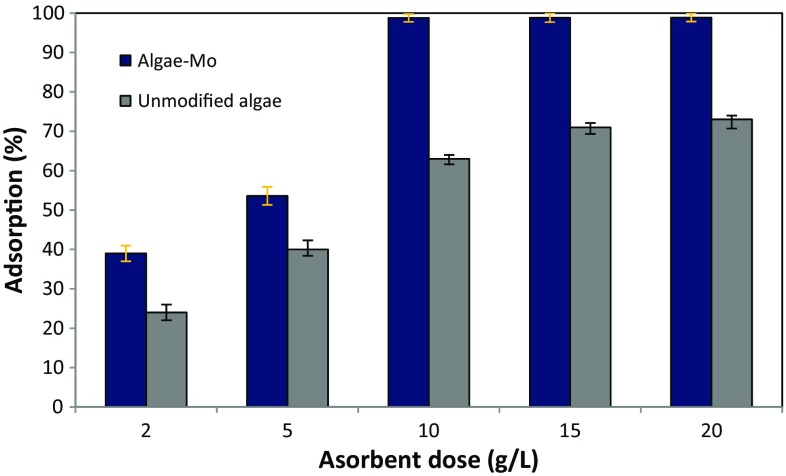
Effect of adsorbent dose on phosphate adsorption (pH:5, phosphate concentration: 50 mg/L, contact time: 60 min)

The influence of dosage of algae–Mo and plain algae on phosphate removal is shown in Fig. 7. By increasing the dose of algae–Mo and plain algae from 2 to 10 g/L, the phosphate adsorption was increased from 39 to 98.7 and 24 to 63%, respectively. This finding may be due to increasing adsorbents’ surface area and thus the availability of more adsorption sites resulting from the increase in dosage (Asgari et al. 2014). It is noticeable from Fig. 7 that in the dosages higher than 10 g/L, no significant changes in the phosphate removal efficiency were observed. The increase of adsorbent dose (more than 10 g/L) would raise the collision probability between different adsorbent particles, which would lead to the overlapping and aggregating of the particles and would reduce the efficient utilization of functional groups on the adsorbent surface, and then influence the phosphate adsorption removal (Ye et al. 2015). Therefore, adsorbent dosage of 10 g/L was selected for the following tests. Generally, the result is supported by other studies (e.g., Liu et al. 2016; Ye et al. 2015), where an increase in phosphate adsorption was observed with an increase in the dose of adsorbent.

### Effect of phosphate concentration and contact time

**Fig. 8:**
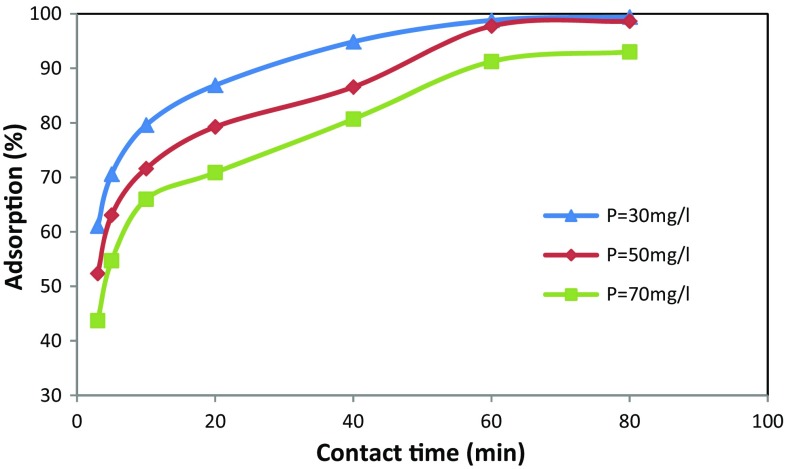
Effect of contact time and initial phosphate concentration on its removal by algae–Mo (adsorbent dose: 10 g/L, pH: 5)

The influence of initial phosphate concentration on the adsorption of phosphate ions onto the algae–Mo was assessed and the results are presented in Fig. 8. From Fig. 8, the trend of phosphate removal was similar for all initial phosphate concentrations. By increasing the contact time, the amount of phosphate removal by the adsorbent increased. As shown in Fig. 8, the percentage of phosphate removal by algae–Mo at contact time of 3 min for initial concentrations of 30, 50, and 70 mg/L was obtained at 43.6, 52.36, and 61.06%, respectively. By increasing the time of contact, the adsorption percentage was remarkably increased at each concentration. For instance, at the contact time of 60 min, the phosphate removal percentage of 98.8, 97.7, and 91.2% were gained for initial concentrations of 30, 50, and 70 mg/L, respectively. It is obvious from the results that the amount of phosphate adsorbed to the adsorbent increased with increasing contact time, and this provided enough time for phosphate in the solution to find free sites to be absorbed (Sun et al. 2014). Thus, the phosphate adsorption onto algae–Mo depends on the initial phosphate concentration and contact time. This result is in agreement with previous literature (Liu et al. 2016; Ogata et al. 2016).

### Temperature effect and thermodynamics

Table 2 illustrates the influence of solution temperature on the adsorption of phosphate by algae–Mo. As observed from Table 2, the percentage of phosphate adsorption onto algae–Mo increased from 91.2 to 100% as the solution temperature was increased from 20 to 40 °C. Thus, the phosphate adsorption at the high temperature will be beneficial to its removal.

**Table 2:** Results of temperature effect and thermodynamic parameters for the adsorption of phosphate ions onto algae–Mo (adsorbent dose 10 g/L, pH 5, phosphate concentration 50 mg/L, contact time 60 min)

Temperature (K)	Adsorption (%)	ln *k*_th_	Δ*G*° (kJ/mol)	Δ*S*° (kJ/mol.*K*)	Δ*H*° (kJ/mol)
293	91.2	19.8	−42.89	−1.74	437.3
298	98.76	35.5	−81.79		
303	99.5	42.8	−106.38		
313	100	51.2	−119.34		

The thermodynamic parameters involved in the adsorption process were obtained and the findings are listed in Table 2. The thermodynamic parameters of equilibrium constant (*k*_th_) (phosphate distribution between solid and liquid phases), standard enthalpy change (Δ*H*°), standard Gibbs free energy (Δ*G*°), and standard entropy change (Δ*S*°) were estimated based on the method described by Rajeswari et al. (2015) and Asgari et al. (2013a). In this method, *k*_th_ was determined by plotting ln(*q*_e_/*C*_e_) against *q*_e_ at different temperatures and extrapolating *q*_e_ to zero (*q*_e_ and *C*_e_ were defined in Eq. 2). The Δ*G*° parameter was calculated from “$\Delta G{^\circ } = - RT\ln k_{\text{th}}$” and Δ*H*° and Δ*S*° parameters were attained from the slope and intercept of plotting ln *k*th against 1/*T*, respectively. These involved using “$\ln k_{th} = \Delta S{^\circ }/R - \Delta H{^\circ }/RT,$” where *R* and *T* are the gas constant and absolute temperature of the working solution, respectively.

The thermodynamic parameters for the phosphate adsorption by the algae–Mo are presented in Table 2. As shown in Table 2, the *K*th value increased with increasing temperature, indicating the endothermic nature of the adsorption (Gao et al. 2013). It was found that declined Δ*G*° from −42.89 to −119.34 kJ/mol when the temperature rose from 20 to 30 °C, which confirm that the adsorption of phosphate ion onto the algae–Mo became more favorable and feasible at high temperature. The negative values of Δ*G*° parameter signify the spontaneity of the adsorption process. The positive value for Δ*H*° specifies that the process is endothermic. Since the value of 432.7 kJ/mol obtained for Δ*H*° is much higher than the range of Δ*H*° values for physical adsorption (i.e., 8.4–41.8 kJ/mol) (Jiang et al. 2016), the adsorption process is specified to have a chemical nature. The negative value of Δ*S*° expresses less randomness of reaction during the phosphate adsorption (Jiang et al. 2016), which can be attributed to the release of water molecules from molybdate accompanied by the coordination between this metal and phosphate. These findings were consistent with the adsorption of phosphate by La–modified tourmaline (Li et al. 2015), natural loess (Jiang et al. 2016), and surfactant loaded hydrothermally synthesized silicate nanoparticles (Bhardwaj et al. 2014).

### Isotherm study

The equilibrium adsorption data obtained in this study were examined according to the three well-known isotherms: Langmuir, Freundlich, and Dubnin–Radushkevich (D–R). The estimated parameters for the adsorption isotherms are listed in Table 3. As understood from these values, it is visible that all isotherm models are fitted to the experimental data. However, based on *R* ^2^ values for the Langmuir model, it is slightly better fitted to experimental data. This result illustrates that a monolayer adsorption, rather than Freundlich heterogeneous surface adsorption, played a main role in the phosphate adsorption process.

**Table 3:** Results of isotherm modeling for adsorption of phosphate onto algae–Mo

Isotherm	Equation	Parameter	Value
Langmuir (*y* = 0.0082*x* + 0.0067)	$q_{\text{e}} = \frac{{Qk_{\text{L}} C_{\text{e}} }}{{1 + k_{\text{L}} C_{\text{e}} }}$	*Q*	149.25
		*k*_L_	0.817
		*R* ^2^	0.9943
Freundlich (*y* = 0.9327*x* + 2.3359)	$q_{\text{e}} = kC_{\text{e}}^{1/n}$	*k*	10.338
		1/*n*	0.9327
		*R* ^2^	0.9817
D–R (*y* = −0.0039*x* + 4.5771)	$\ln q_{\text{e}} = \ln q_{\text{m}} - K_{\text{DR}} \varepsilon^{2}$	*q*_m_	97.23
		*K*_DR_	4E−09
		*E*	11.2
		*R* ^2^	0.9371

*q*_*m*_ maximum adsorption capacity (mg/g), *k*_L_ Langmuir constant (L/mg), *k* Freundlich constant, *n* Freundlich constant (mg/g(L/mg)^1/*n*^), *K*_*DR*_ *D*–*R* constant (mol^2^/kJ^2^), *ε* Polanyi potential (J/mol), *E* free energy (kJ/mol)

Using the Langmuir isotherm model (Table 3), the estimated maximum adsorption capacity (*Q*) of algae–Mo was 149.25 mg/g, which indicated that the adsorption capacity of algae–Mo was much greater than those recently obtained by other adsorbents for removal of phosphate from aqueous solution (Hamoudi and Belkacemi 2013; Huang et al. 2015; Liu and Zhang 2015), with adsorption capacities ranging from 17.2 to 60.6 mg/g. Thus, the algae–Mo studied in this paper was highly competitive when compared to most of the adsorbents examined for the phosphate adsorption in the recent literature. Moreover, the base material of the algae–Mo is easily available in the algal blooms, making the cost of consumables and the preparation price similar to other waste-based adsorbents.

Also, as shown in Table 3, the values of heterogeneity factor (1/*n*) are less than unity, which proves that algae–Mo is an appropriate and beneficial adsorbent for phosphate adsorption (Wang et al. 2014).

Referring to *R* ^2^ values in Table 3, it is clear that the D–R isotherm (with *R* ^2^ = 0.9371) had also a good correlation with the experimental data. The amount of free energy (*E*) in phosphate adsorption by the algae–Mo is estimated by the D–R model and was found to be 11.2 kJ/mol. The value of E reveals that the chemisorption mechanism took place as it fell within the range of 8–16 kJ/mol (Asgari et al. 2013a; Khademi et al. 2015).

### Kinetic study

**Fig. 9:**
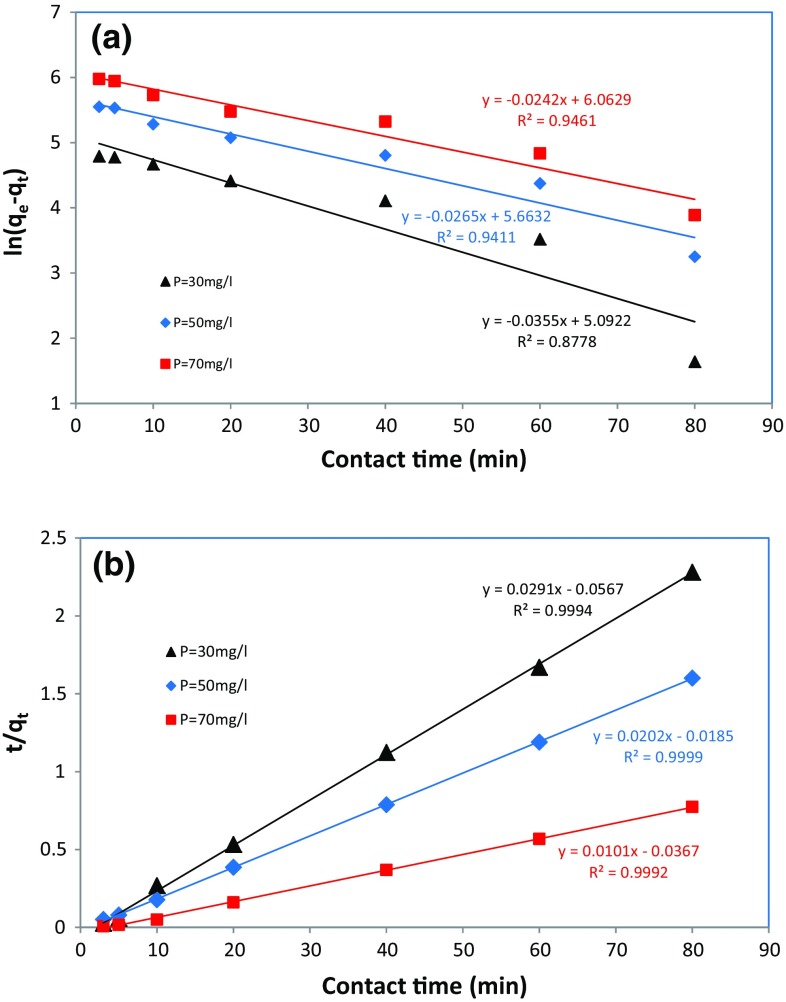
Kinetic models of **a** pseudo-first-order model, **b** pseudo-second-order model (adsorbent dose 10 g/L, pH 5)

Phosphate adsorption experimental data were fitted to the most well known of kinetic models, namely the pseudo-first-order and the pseudo-second-order model. The correlation coefficient (*R* ^2^) and kinetic information obtained from the aforementioned models (displayed in Fig. 9) for the different initial concentrations of phosphate have been presented in Table 4. The fitted linear regression plots (Fig. 9) and the results of fitting models (Table 4) showed that the experimental data obtained from the phosphate adsorption by algae–Mo had the best fit with the pseudo-second-order model for the investigated concentration (30, 50, and 70 mg/L) with the higher coefficients of determination (*R* ^2^ > 0.999) than those of the pseudo-first-order model. Therefore, the rate of phosphate adsorption onto the algae–Mo is of pseudo-second order, suggesting that the adsorption of phosphate onto the adsorbent is influenced by both the adsorbate and the adsorbent concentrations under the investigated conditions (Asgari et al. 2013b). In addition, as can be seen from Table 4, the difference between the experimental *q*_e_ values and the model-calculated *q*_e_ is very small (less than 2%), reaffirming the high correlation of adsorption to the pseudo-second-order model. The decrease of *k*_2_ values with increase in the initial phosphate concentration showed a good concordance between the experimental data and the pseudo-second-order kinetic model. A review of the recently published literature (Hamoudi and Belkacemi 2013; Li et al. 2014; Riahi et al. 2013) on the phosphate adsorption showed that most researchers reported that the pseudo-second-order model satisfactory fits the experimental data.

**Table 4:** Results of kinetic study for the adsorption of phosphates onto algae–Mo (Khademi et al. 2015)

Parameter	Initial phosphate concentrations (mg/L)		
	30	50	70
Experimental *q*_e_	19.23	51.4	101.35
Pseudo-first order			
*k*_1_	0.0355	0.0265	0.0242
*q*_e_	162.75	288.07	429.62
*R* ^2^	0.8778	0.9411	0.9461
Pseudo-second order			
*k*_2_	0.065	0.022	0.003
*q*_e_	17.64	49.5	99
*R* ^*2*^	0.9994	0.9999	0.9992

Pseudo-first order: *q*_*t*_ = *q*_e_[1−exp(−*k*_1_ *t*)]

Pseudo-second order: $q_{\text{t}} = \frac{{k_{2} q_{\text{e}}^{ 2} t}}{{1 + k_{2} q_{\text{e}} t}}$

*k*_1_ rate constant of pseudo-first-order model (1/min), *k*_2_ rate constant of pseudo-second-order model (mg/g min), *q*_t_ adsorbed amount at any time (mg/g), *q*_e_ adsorbed amount at equilibrium (mg/g)

### Recycling of the adsorbent

**Fig. 10:**
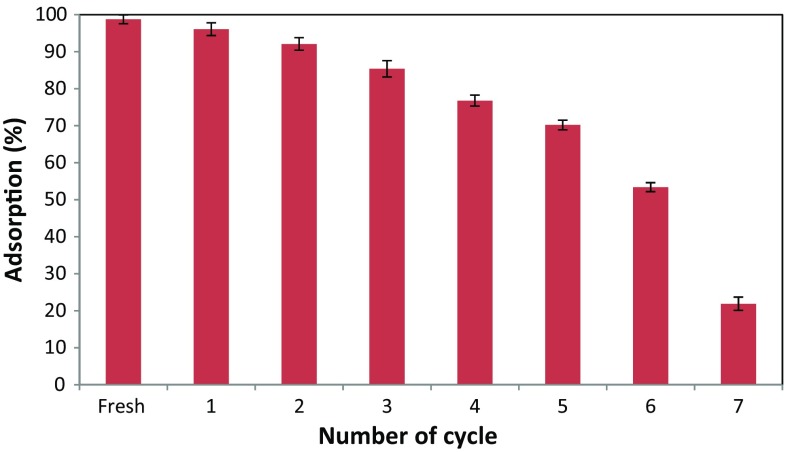
Effects of adsorbent reuse times on phosphate adsorption (adsorbent dose: 10 g/L, pH: 5, phosphate concentration: 50 mg/L, contact time: 60 min)

To assess the recyclability of the algae–Mo in the phosphate adsorption, an experimental phase was carried out. To do this, the recyclability of the adsorbent was tested in seven consecutive cycles under identical conditions (pH 5, initial phosphate concentration of 50 mg/L, contact time 60 min, and solution temperature 25 °C) and the adsorbent was being recycled from the previous test without any modifications. The phosphate removal efficiency was determined after each test, and the results are presented in Fig. 10. As indicated, the algae–Mo preserved its adsorption capability after the sixth reuse (with efficiency >50%); thus, the algae–Mo is a stable adsorbent for attenuating phosphate-containing wastewaters. Conclusively, the recyclable properties of algae–Mo adsorbent support its commercial use. The cycle number (i.e., 6 cycles) of reusability of the adsorbent is more than that obtained by nanostructured iron(III)–copper(II) binary oxides studied by Li et al. (2014).

### Real wastewater treatment (2 samples)

Two wastewater samples were collected from the hospital wastewater treatment plant and an urban wastewater before entering the Persian Gulf (as sophisticated matrix) for treatment by the algae–Mo; their specifications are presented in Table 5. The real samples were treated using algae–Mo with optimized conditions to confirm its practical applicability. The original pH of both the collected samples was not adjusted to a desired amount. After treating the hospital and urban wastewaters with algae–Mo, the phosphate concentration was reduced by 86.8 and 90%, respectively. The high removal of phosphate from urban wastewater with high amount of TDS (as sophisticated matrix) could be justified with low initial level of phosphate. Other contaminants such as NO_3_^−^, BOD_5_, and COD were decreased relatively after treatment with algae–Mo. From the above decrease in concentration values, the algae–Mo proves to be an excellent adsorbent for removing phosphate ions from phosphate-containing wastewaters.

**Table 5:** Real wastewater treatment by algae–Mo (adsorbent dose: 10 g/L, pH: as per original, contact time: 60 min)

Properties	Sample 1: hospital wastewater		Sample 2: urban wastewater before entering seawater	
	Concentration before treatment (mg/L)	Concentration after treatment (mg/L)	Concentration before treatment (mg/L)	Concentration after treatment (mg/L)
PO_4_^3−^	23.6	3.1	12.1	1.2
NO_3_^−^	11	6	69	34
Cl^−^	112.5	24	212.5	54
SO_4_^2−^	24	20	124	120
Total hardness	346	338	458	438
TDS	672	660	1060	905
BOD_5_	267.5	258	185	173.4
COD	532	511	364	355
pH	6.5	7.25	7.7	7.9

### Molybdate leaching from algae–Mo

The risk of Mo^2+^ leakage from algae–Mo during phosphate adsorption is important from practical applicability point of view. The releasing behavior of Mo^2+^ was tested by measuring Mo^2+^ concentration in the solution under pH values of 3–9. Within the pH range of 3–9, the Mo^2+^ concentrations in the treated solutions were almost zero, indicating good stability of the intercalated Mo^2+^ in the algae of *S. angustifolium*. The results are in conformity with those obtained in the EDS study, which showed that only 0.04% of molybdate leached the treated solution.

## Conclusion

In this study, the brown algae *S. angustifolium* were modified by molybdate ion during facile cultivation to produce a new adsorbent, namely algae–Mo. The characteristics of algae–Mo were studied using the FTIR, EDS, SEM, and BET methods. The phosphate adsorption performance of the algae–Mo was explored in a batch mode. The effects of pH, initial phosphate concentration, algae–Mo dose, and temperature on the phosphate adsorption efficiency were investigated. The maximum phosphate adsorption (98.7%) was attained at pH, algae–Mo dose, and temperature of 5, 10 g/L, and 50 °C, respectively. Thermodynamic assessment revealed that the nature of phosphate adsorption by algae–Mo was feasible, spontaneous, and endothermic. The Langmuir isotherm model fit the experimental data well with a maximum monolayer adsorption capacity of 149.25 mg/g. The adsorption equilibrium data were adequately fitted to a pseudo-second-order kinetic model (*R* ^2^ = 0.9999). Overall, the findings obtained in this study show that the novel algae–Mo adsorbent is promising and could be repeatedly used without any concern about leaching of Mo^2+^ for the phosphate ion removal from wastewaters.

## References

[CR1] Asgari G, Ramavandi B, Farjadfard S (2013). Abatement of azo dye from wastewater using bimetal-chitosan. Sci World J.

[CR2] Asgari G, Ramavandi B, Rasuli L, Ahmadi M (2013). Cr (VI) adsorption from aqueous solution using a surfactant-modified Iranian zeolite: characterization, optimization, and kinetic approach. Desalin Water Treat.

[CR3] Asgari G, Ramavandi B, Sahebi S (2014). Removal of a cationic dye from wastewater during purification by Phoenix dactylifera. Desalin Water Treat.

[CR4] Bhardwaj D, Sharma P, Sharma M, Tomar R (2014). Removal and slow release studies of phosphate on surfactant loaded hydrothermally synthesized silicate nanoparticles. J Taiwan Inst Chem Eng.

[CR5] Biswas BK, Inoue K, Ghimire KN, Harada H, Ohto K, Kawakita H (2008). Removal and recovery of phosphorus from water by means of adsorption onto orange waste gel loaded with zirconium. Biores Technol.

[CR6] Carvalho WS, Martins DF, Gomes FR, Leite IR, da Silva LG, Ruggiero R, Richter EM (2011). Phosphate adsorption on chemically modified sugarcane bagasse fibres. Biomass Bioenergy.

[CR7] Federation WE, Association APH (2005). Standard methods for the examination of water and wastewater.

[CR8] Fooladvand M, Ramavandi B (2015). Adsorption potential of NH_4_Br-soaked activated carbon for cyanide removal from wastewater. Indian J Chem Technol.

[CR9] Gao S, Wang C, Pei Y (2013). Comparison of different phosphate species adsorption by ferric and alum water treatment residuals. J Environ Sci.

[CR10] Hamoudi S, Belkacemi K (2013). Adsorption of nitrate and phosphate ions from aqueous solutions using organically-functionalized silica materials: kinetic modeling. Fuel.

[CR11] Huang W, Zhu Y, Tang J, Yu X, Wang X, Li D, Zhang Y (2014). Lanthanum-doped ordered mesoporous hollow silica spheres as novel adsorbents for efficient phosphate removal. J Mater Chem A.

[CR12] Huang W, Chen J, He F, Tang J, Li D, Zhu Y, Zhang Y (2015). Effective phosphate adsorption by Zr/Al-pillared montmorillonite: insight into equilibrium, kinetics and thermodynamics. Appl Clay Sci.

[CR13] Ismail ZZ (2012). Kinetic study for phosphate removal from water by recycled date-palm wastes as agricultural by-products. Inter J Environ Stud.

[CR14] Jiang S, Wang X, Yang S, Shi H (2016). Characteristics of simultaneous ammonium and phosphate adsorption from hydrolysis urine onto natural loess. Environ Sci Poll Res.

[CR15] Johir M, Pradhan M, Loganathan P, Kandasamy J, Vigneswaran S (2015). Phosphate adsorption from wastewater using zirconium (IV) hydroxide: kinetics, thermodynamics and membrane filtration adsorption hybrid system studies. J Environ Manag.

[CR16] Johir MA, Pradhan M, Loganathan P, Kandasamy J, Vigneswaran S (2016). Phosphate adsorption from wastewater using zirconium (IV) hydroxide: kinetics, thermodynamics and membrane filtration adsorption hybrid system studies. J Environ Manag.

[CR17] Khademi Z, Ramavandi B, Ghaneian MT (2015). The behaviors and characteristics of a mesoporous activated carbon prepared from *Tamarix hispida* for Zn (II) adsorption from wastewater. J Environ Chem Eng.

[CR18] Lalley J, Han C, Li X, Dionysiou DD, Nadagouda MN (2016). Phosphate adsorption using modified iron oxide-based sorbents in lake water: kinetics, equilibrium, and column tests. Chem Eng J.

[CR19] Li G, Gao S, Zhang G, Zhang X (2014). Enhanced adsorption of phosphate from aqueous solution by nanostructured iron (III)–copper (II) binary oxides. Chem Eng J.

[CR20] Li G, Chen D, Zhao W, Zhang X (2015). Efficient adsorption behavior of phosphate on La-modified tourmaline. J Environ Chem Eng.

[CR21] Liu X, Zhang L (2015). Removal of phosphate anions using the modified chitosan beads: adsorption kinetic, isotherm and mechanism studies. Powder Technol.

[CR22] Liu Q, Hu P, Wang J, Zhang L, Huang R (2016). Phosphate adsorption from aqueous solutions by Zirconium (IV) loaded cross-linked chitosan particles. J Taiwan Inst Chem Eng.

[CR23] Lu J, Liu H, Zhao X, Jefferson W, Cheng F, Qu J (2014). Phosphate removal from water using freshly formed Fe–Mn binary oxide: adsorption behaviors and mechanisms. Colloids Surf A Physicochem Eng Asp.

[CR24] Lu J, Liu D, Hao J, Zhang G, Lu B (2015). Phosphate removal from aqueous solutions by a nano-structured Fe–Ti bimetal oxide sorbent. Chem Eng Res Des.

[CR25] Lǚ J, Liu H, Liu R, Zhao X, Sun L, Qu J (2013) Adsorptive removal of phosphate by a nanostructured Fe–Al–Mn trimetal oxide adsorbent. Powder Technol 233:146–154

[CR26] Ma L, Feng S, Reidsma P, Qu F, Heerink N (2014). Identifying entry points to improve fertilizer use efficiency in Taihu Basin, China. Land Use Policy.

[CR27] Mallampati R, Valiyaveettil S (2013). Apple peels—a versatile biomass for water purification?. ACS Appl Mater Interfaces.

[CR28] Nguyen T, Ngo H, Guo W, Zhang J, Liang S, Tung K (2013). Feasibility of iron loaded ‘okara’ for biosorption of phosphorous in aqueous solutions. Biores Technol.

[CR29] Nguyen T, Ngo H, Guo W, Nguyen T, Zhang J, Liang S, Chen S, Nguyen N (2014). A comparative study on different metal loaded soybean milk by-product ‘okara’ for biosorption of phosphorus from aqueous solution. Biores Technol.

[CR30] Nguyen T, Ngo H, Guo W, Zhang J, Liang S, Lee D, Nguyen P, Bui X (2014). Modification of agricultural waste/by-products for enhanced phosphate removal and recovery: potential and obstacles. Biores Technol.

[CR31] Ogata F, Imai D, Toda M, Otani M, Kawasaki N (2016). Properties of a novel adsorbent produced by calcination of nickel hydroxide and its capability for phosphate ion adsorption. J Ind Eng Chem.

[CR32] Rajeswari A, Amalraj A, Pius A (2015). Removal of phosphate using chitosan-polymer composites. J Environ Chem Eng.

[CR33] Ramavandi B, Rahbar A, Sahebi S (2016). Effective removal of Hg^2+^ from aqueous solutions and seawater by Malva sylvestris. Desalin Water Treat.

[CR34] Riahi K, Chaabane S, Thayer BB (2013) A kinetic modeling study of phosphate adsorption onto Phoenix dactylifera L. date palm fibers in batch mode. J Saudi Chem Soc **(in press)**

[CR35] Singh NK, Raghubanshi AS, Upadhyay AK, Rai UN (2016). Arsenic and other heavy metal accumulation in plants and algae growing naturally in contaminated area of West Bengal, India. Ecotoxicol Environ Safe.

[CR36] Sun X, Imai T, Sekine M, Higuchi T, Yamamoto K, Kanno A, Nakazono S (2014). Adsorption of phosphate using calcined Mg 3–Fe layered double hydroxides in a fixed-bed column study. J Ind Eng Chem.

[CR37] Wang Z, Nie E, Li J, Yang M, Zhao Y, Luo X, Zheng Z (2012). Equilibrium and kinetics of adsorption of phosphate onto iron-doped activated carbon. Environ Sci Poll Res.

[CR38] Wang Z, Shi M, Li J, Zheng Z (2014). Influence of moderate pre-oxidation treatment on the physical, chemical and phosphate adsorption properties of iron-containing activated carbon. J Environ Sci.

[CR39] Ye J, Cong X, Zhang P, Hoffmann E, Zeng G, Wu Y, Zhang H, Fan W (2015). Phosphate adsorption onto granular-acid-activated-neutralized red mud: parameter optimization, kinetics, isotherms, and mechanism analysis. Water Air Soil Poll.

[CR40] Zhang J, Shan W, Ge J, Shen Z, Lei Y, Wang W (2011). Kinetic and equilibrium studies of liquid-phase adsorption of phosphate on modified sugarcane bagasse. J Environ Eng.

